# A New Butyrate Releaser Exerts a Protective Action against SARS-CoV-2 Infection in Human Intestine

**DOI:** 10.3390/molecules27030862

**Published:** 2022-01-27

**Authors:** Lorella Paparo, Maria Antonia Maglio, Maddalena Cortese, Cristina Bruno, Mario Capasso, Erika Punzo, Veronica Ferrucci, Vito Alessandro Lasorsa, Maurizio Viscardi, Giovanna Fusco, Pellegrino Cerino, Alessia Romano, Riccardo Troncone, Massimo Zollo

**Affiliations:** 1 Dipartimento di Scienze Mediche Translazionali, Università degli Studi di Napoli “Federico II”, 80131 Napoli, Italy; mariantonia.maglio@unina.it (M.A.M.); Maddalenacortese95@hotmail.com (M.C.); cristinabruno88@libero.it (C.B.); pun.erika@gmail.com (E.P.); troncone@unina.it (R.T.); 2 CEINGE—Advanced Biotechnologies s.c.ar.l., Università degli Studi di Napoli “Federico II”, 80131 Napoli, Italy; mario.capasso@unina.it (M.C.); veronica.ferrucci@libero.it (V.F.); lasorsa.alessandro@gmail.com (V.A.L.); romanoa@ceinge.unina.it (A.R.); massimo.zollo@unina.it (M.Z.); 3 Dipartimento di Medicina Molecolare e Biotecnologie Mediche (DMMBM), Università degli Studi di Napoli “Federico II”, 80131 Napoli, Italy; 4 DAI Medicina di Laboratorio e Trasfusionale, AOU Azienda Ospedaliera, Università degli Studi di Napoli “Federico II”, 80131 Napoli, Italy; maurizioviscardi@gmail.com (M.V.); giovannafusco@gmail.com (G.F.); pellegrinocerino@gmail.com (P.C.)

**Keywords:** COVID-19, viral infection, transmembrane protease serine 2, angiotensin-converting enzyme-2, intestinal models

## Abstract

Butyrate is a major gut microbiome metabolite that regulates several defense mechanisms against infectious diseases. Alterations in the gut microbiome, leading to reduced butyrate production, have been reported in patients with severe acute respiratory syndrome coronavirus 2 (SARS-CoV-2) infection. A new butyrate releaser, useful for all the known applications of butyrate, presenting physiochemical characteristics suitable for easy oral administration, (*N*-(1-carbamoyl-2-phenyl-ethyl) butyramide (FBA), has been recently developed. We investigated the protective action of FBA against SARS-CoV-2 infection in the human small intestine and enterocytes. Relevant aspects of SARS-CoV-2 infection were assessed: infectivity, host functional receptor angiotensin-converting enzyme-2 (*ACE2*), transmembrane protease serine 2 (*TMPRSS2*), neuropilin-1 (*NRP1*), pro-inflammatory cytokines expression, genes involved in the antiviral response and the activation of Nf-kB nuclear factor (erythroid-derived 2-like) 2 (Nfr2) pathways. We found that FBA positively modulates the crucial aspects of the infection in small intestinal biopsies and human enterocytes, reducing the expression of *ACE2*, *TMPRSS2* and *NRP1*, pro-inflammatory cytokines interleukin *(IL)-15*, monocyte chemoattractant protein-1 (*MCP-1*) and *TNF-α*, and regulating several genes involved in antiviral pathways. FBA was also able to reduce the number of SARS-CoV-2-infected cells, and *ACE2*, *TMPRSS2* and *NRP1* expression. Lastly, through the inhibition of Nf-kB and the up-regulation of Nfr2, it was also able to reduce the expression of pro-inflammatory cytokines *IL-15*, *MCP-1* and *TNF-α* in human enterocytes. The new butyrate releaser, FBA, exerts a preventive action against SARS-CoV-2 infection. It could be considered as an innovative strategy to limit COVID-19.

## 1. Introduction

There is a global pandemic of coronavirus disease 2019 (COVID-19), caused by severe acute respiratory syndrome coronavirus 2 (SARS-CoV-2) [1]. The gastrointestinal tract is a major target organ for SARS-CoV-2 infection, with many patients presenting with gastrointestinal symptoms, such as nausea/vomiting, diarrhea and abdominal pain. These symptoms are even more frequent in severe COVID-19 patients [2,3].

The angiotensin-converting enzyme-2 (ACE2) and the transmembrane protease serine-2 (TMPRSS2) are two key molecules in SARS-CoV-2 infection that are highly expressed in the gastrointestinal tract [4,5]. In addition, neuropilin-1 (NRP1), a SARS-CoV-2 co-receptor, enhances the ability of SARS-CoV-2 to enter host cells and potentiates the infectivity of the virus [5,6,7]. As consequence of SARS-CoV-2 infection, host defenses launch a counterattack, releasing inflammatory cytokines, resulting in a “cytokine storm” [8]. The “cytokine storm” attempts to eliminate the virus, but, in the process, collateral damage occurs in several tissues, mainly in the gastrointestinal and respiratory tracts [9]. The inflammation induced by SARS-CoV-2 infection is also reflected by high blood levels of several inflammatory cytokines [10]. The increase in the number of inflammatory cytokines in severe COVID-19 patients is considered to be a predictor of higher mortality rate [11]. Accordingly, some successes have been reported when treating COVID-19 patients with anti-inflammatory agents [10]. Of note since now, not many drugs are known to act against the immune response that is actively induced by SARS-CoV-2 [12]. Thus, recently, phosphate food additives, including long chain PolyP, have been proven at low doses to inhibits SARS-CoV-2 infection and “the cytokine storm” [13].

Emerging evidence reports the presence of gut microbiome dysbiosis in COVID-19 patients, with a reduction of short chain fatty acids (SCFAs)-producing bacteria, such as *Faecalibacterium prausnitzii* [14,15,16]. The alteration of the gut microbiome structure (and function) seems to be higher in more severe COVID-19 patients [15]. Butyrate is major SCFAs produced by the gut microbiome, and it plays a pivotal role in human health [11]. Butyrate acts as key modulator for defense against viral infections through the modulation of several immune and non-immune mechanisms [17,18,19]. Butyrate is also able to exert a potent anti-inflammatory action in the gastrointestinal tract [20,21]. Conceptually, supplementing patients infected with SARS-CoV-2 with butyrate could serve as a helpful adjunct in preventing and treating human tissue inflammation induced by the infection. Unfortunately, the negative organoleptic profile characterized by an extremely offensive odor and taste limits the use of butyrate in human medicine and nutrition [22].

*N*-(1-carbamoyl-2-phenyl-ethyl) butyramide (FBA) is a novel butyric acid releaser, which shows physicochemical characteristics suitable for easy oral administration, being totally free of the unpleasant organoleptic properties that characterize butyrate [22]. The proposed use of FBA would accomplish two things: the direct release of butyrate into the intestinal tract, which contains one of the highest concentrations of SARS-CoV-2 receptors and increase butyrate availability for the body.

Therefore, in this study, we investigated the direct effect of FBA on the main players of SARS-CoV-2 infection: *ACE2*, *TMPRSS2* and *NRP1* [4,5,6,7], and on other key genes related to anti-viral pathways, as well as on inflammatory cytokines response in the gut.

Our results highlighted the protective role of this new butyrate releaser against host infection and inflammatory response, thus providing a scientific basis for the use of FBA against COVID-19.

## 2. Results

### 2.1. FBA Reduces the Expression of the Main Molecular Mediators of SARS-CoV-2 Infection and Inflammatory Cytokines in Ex Vivo and In Vitro Intestinal Models

In a first set of experiments, we investigated the direct effect of FBA on the expression of the main molecular players of SARS-CoV-2 infection: *ACE2*, *TMPRSS2*, *NRP1* [4,5,6,7] and inflammatory cytokines.

**Figure 1:**
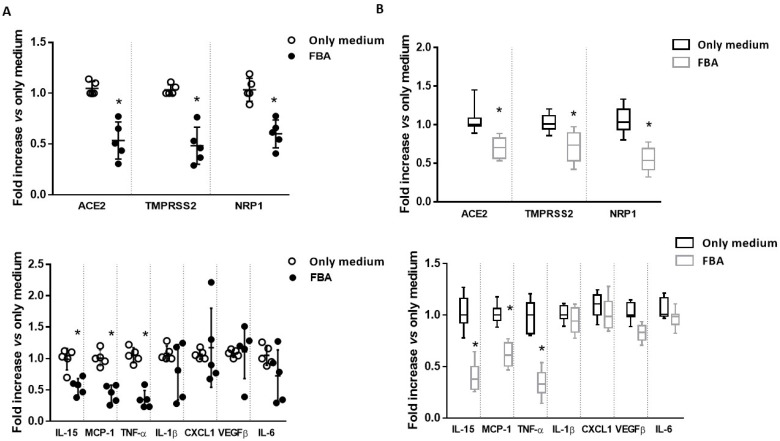
Effect of FBA on the main molecular players of SARS-CoV-2 infection and inflammatory cytokines expression in ex vivo and in vitro models. (**A**) Small intestinal biopsies from 5 healthy subjects were treated with 2 mM FBA for 24 h. Cells were collected for RNA extraction. FBA was able to reduce *ACE2*, *TMPRSS2* and *NRP1* (panel above) and *IL-15*, *MCP-1*, and *TNF-α* expression (panel below). (**B**) Caco-2 cells were treated with 2 mM FBA for 24 h. Cells were collected for RNA extraction. The treatment with FBA reduced *ACE2*, *TMPRSS2* and *NRP1* (panel above) and *IL-15*, *MCP-1*, and *TNF-α* expression (panel below). Data represent the means with ±SD of 3 independent experiments, each performed in triplicate. Data were analyzed using a paired *t*-test. * *p* < 0.05 vs. only medium.

We incubated human small intestine biopsy samples, collected from five healthy subjects, with FBA. We found that *ACE2*, *TMPRSS2* and *NRP1* expression, the cellular mediators that facilitate SARS-CoV-2 entry into host cells, were significantly reduced by FBA. Furthermore, FBA elicited a significant reduction in the expression of the following inflammatory cytokines: interleukin *(IL)-15*, monocyte chemoattractant protein-1 (*MCP-1*) and tumor necrosis factor (TNF)-α (Figure 1A).

Then, we moved to another set of experiments performed on the human enterocytes cell line (Caco-2 cells) to investigate the reproducibility of these effects in a different experimental model. We obtained similar results: FBA was able to significantly reduce *ACE2*, *TMPRSS2* and *IL-15*, as well as *MCP-1* and *TNF-α* expression in human enterocytes (Figure 1B).

### 2.2. FBA Regulates the Expression of Genes Related to Anti-Viral Pathways

**Figure 2:**
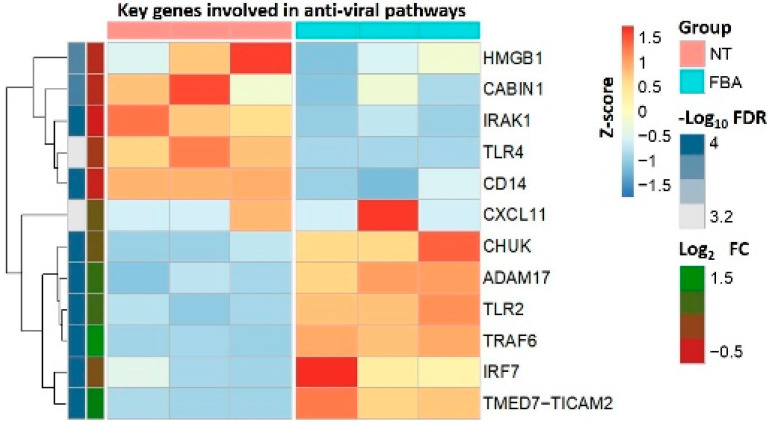
Effects of FBA on several genes involved in the anti-viral pathways. Heatmap reporting the average relative expression of different genes between untreated cells (NT) and 2 mM FBA-stimulated human enterocytes (administered FBA for 24 h). Differentially expressed genes were (i) those with a Log2 fold change above 0.3 or below −0.3, and an adjusted *p*-value < 0.05 (here shown as −Log10 FDR).

To determine the effects of FBA on the transcriptional program of other key genes related to anti-viral pathways in human enterocytes, we performed high-throughput RNA sequencing. Among the genes reported in the heatmap (Figure 2), we found that FBA reduced the expression of several pivotal genes involved in anti-viral function (Supplementary Materials: Table S1): high-mobility group protein-1 (HMGB1), crucial mediator for SARS-CoV-2 replication at the post-entry phase [23]; interleukin-1 receptor-associated kinase 1 (*IRAK1*), responsible for the induction of the inflammatory cytokines and chemokines response associated with morbidity and mortality in COVID-19 patients [24]; the cluster of differentiation-14 (*CD14*), a possible target to control inflammation and organ dysfunction in SARS-CoV-2 infection [25]; *TLR4*, a receptor able to bind the SARS-CoV-2 spike protein, leading to the cytokine storm in severe COVID-19 patients [26]. In contrast, FBA elicited an up-regulation of the genes involved in expression in the Toll-like receptor (TLR) signaling pathway, an innate immune system targeting viruses: *CHUK*, an inhibitor of Nf-kB [27]; *Adam17*, a metallopeptidase involved in the shedding of ACE2 [28]; *TRAF6*, a negative regulator of cytokine storms [29]; interferon regulatory factor-7 (*Irf7*), which is inhibited in the gastrointestinal tract of COVID-19 patients [30].

### 2.3. FBA Prevents the SARS-CoV-2 Infection through Nf-Kb and Nuclear Factor Erythroid 2-Related factor 2 (Nfr2) Regulation

In the second set of experiments, we investigated the direct effect of FBA on infectivity, on *ACE2*, *TMPRSS2* and *NRP1* and on inflammatory cytokines expression in the human enterocytes infected by WT SARS-CoV-2.

**Figure 3:**
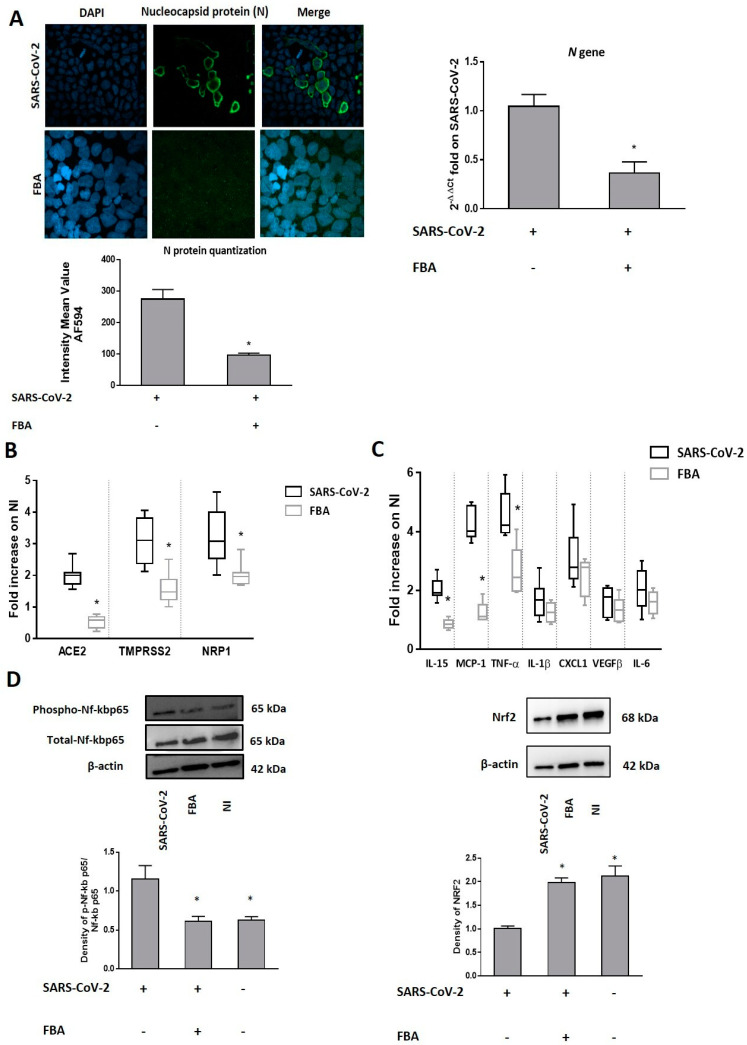
FBA prevented SARS-CoV-2 infection through Nf-Kb and nuclear factor (erythroid-derived 2-like) 2 (Nfr2) regulation. (**A**) Caco-2 cells were infected with 0.1 MOI of WT SARS-CoV-2 that was pretreated with 2 mM FBA for 24 h. Cells were collected for RNA and protein extraction and processed for immunofluorescence. The N protein was visualized using N Alexa Fluor-594 (green) and nuclei were stained with DAPI (blue). Cells were observed through confocal microscopy (**Left** panel). SARS-CoV-2 infection in Caco-2 cells was confirmed by the quantification of N proteins by immunofluorescence staining. The pretreatment with FBA resulted in a reduction in the number of SARS-CoV-2-infected enterocytes. Representative imagines are reported in Figure 3A. Scale bar, 20 µm. (**Right** panel) The quantification of the N gene of viral RNA by RT-PCR analysis. The pretreatment with FBA inhibited N gene transcription in SARS-CoV-2-infected cells. (**B**) Pre-incubation with FBA significantly down-regulated *ACE2*, *TMPRSS2* and *NRP1* expression in Caco-2 cells exposed to WT SARS-CoV-2. (**C**) Pre-incubation with FBA significantly reduced the *IL-15*, *MCP-1* and *TNF-α* expression in Caco-2 cells exposed to WT SARS-CoV-2. (**D**) Pre-incubation with FBA inhibited Nf-kB activation and increased antioxidant nuclear factor Nfr2. The amounts of these proteins and of β-actin were measured via Western blot. The histogram below shows optical density of the proteins, obtained with Image Lab software. The relative quantification of proteins was normalized against β-actin proteins and was calculated using the ratio between phosphorylated and total proteins. The figure shows representative images of three experiments that are qualitatively similar. The expression data are normalized against non-infected cells (NI). “+” or “–“ mean the presence or the absence of a particular compound. Data represent the means with ±SD of 3 independent experiments, each performed in triplicate. Data were analyzed using a paired *t*-test. * *p* < 0.05.

We observed that FBA was able to inhibit the virus entry, as demonstrated by immunofluorescence staining and RT-PCR for the N viral protein (Figure 3A).

The expression of the main molecular mediators of SARS-CoV-2 infection, *ACE2*, *TMPRSS2* and *NRP1*, was significantly down-regulated by FBA in WT SARS-CoV-2-infected cells (Figure 3B). Furthermore, we evaluated the expression of the most important inflammatory cytokines/chemokines commonly observed in COVID-19 patients [31], and we found that FBA was able to reduce the expression of *IL-15*, *MCP-1* and *TNF-α* in human enterocytes exposed to WT SARS-CoV-2 (Figure 3C).

To investigate the mechanisms by which FBA reduces the inflammatory cytokines response, we focused on Nf-kB activation and on antioxidant transcription factor Nfr2, both of which are involved in the regulation of inflammatory responses. It is known that SARS-CoV-2 infection triggers endoplasmic reticulum stress responses in the infected cells associated with reduced Nfr2 and increased levels of reactive oxygen species (ROS) [32]. These mechanisms trigged the “cytokine storm” through Nf-kB activation, a negative regulator of Nfr2 [32,33,34]. We found that FBA reduced Nf-kB activation and increased Nfr2 protein levels in human enterocytes exposed to WT SARS-CoV-2, leading to an antioxidant cellular state (Figure 3D).

## 3. Discussion

The gastrointestinal tract is a target organ for SARS-CoV-2 infection [2,3,35]. Gut microbiome alterations, with the depletion of butyrate-producing bacteria, have been documented in SARS-CoV-2 infection, and in most patients with severe COVID-19 [14,15,16]. These data are not unexpected, since butyrate exerts a pivotal role in protecting the human body against infections and inflammation [21,36,37].

We explored the potential of FBA, a new butyrate releaser, against SARS-CoV-2 infection in human intestine. FBA is a palatable compound with identical functionality to natural butyrate, and it acts as a pure butyrate releaser into the human intestine, maintaining the same pharmacokinetic properties and safety profile of butyrate [22]. Previous evidence confirmed that FBA exerts a potent anti-inflammatory action in different experimental models with a similar extent of butyrate [38,39].

We found that FBA was able to modulate the main players of SARS-CoV-2 infection: *ACE2*, *TMPRSS2* and *NRP-1* [4,5,6,7] and inflammatory cytokines *IL-15*, *MCP-1* and *TNF-α* in the human intestine and enterocytes.

The results from RNA sequencing showed that FBA is also able to modulate several other mechanisms involved in SARS-CoV-2 infection, such as *HMGB1*, which is involved in virus replication [23], *IRAK1* [24], *CD14*, a target to control the inflammation of COVID-19 [25] and *TLR4*, another receptor for the SARS-CoV-2 spike protein [26].

FBA was also able to up-regulate the expression of genes involved in the TLR signaling pathway, such as *CHUK*, an inhibitor of Nf-kB [27], *TRAF6*, a mediator of the inflammatory response [28] and *Irf7*, which is correlated with COVID-19 gastrointestinal symptoms [29].

Our data are well in line with the results of previous studies, demonstrating that butyrate positively modulates *ACE2* and *TMPRSS2* and the inflammatory response, and influences the expression of other anti-viral genes at the intestinal level, through an increase in acetylation of histone-H3 [40,41].

The protective effects of FBA against SARS-CoV-2 infection were confirmed also in human enterocytes. We found that this compound was able to significantly reduce the number of infected cells, as demonstrated by the reduction of N viral protein positive cells and their transcripts. The binding affinity of the SARS-CoV-2 spike protein and ACE2, and its cleaving through TMPRSS2, represent the major determinants of the SARS-CoV-2 replication rate [4]. SARS-CoV-2 infection is potentiated by the co-receptor NRP1, which enhances the ability of SARS-CoV-2 to enter host cells [6,7]. The blocking of viral receptor-binding domains represents a key step in anti-viral approaches [42]. We found that FBA was able to reduce *ACE2*, *TMPRSS2* and *NRP1* expression, preventing SARS-CoV-2 entry into human enterocytes.

The gastrointestinal tract contributes to the cytokine storm in COVID-19 patients [43]. Gut microbiota dysbiosis correlates with the high plasma concentrations of several inflammatory cytokines in COVID-19 patients [16]. The modulation of these cytokines is a relevant target to improve COVID-19 outcomes [44]. We observed that FBA was able to reduce *MCP-1*, *IL-15* and *TFN-α*. Among these cytokines, MCP-1 and TNF-α could be promising biomarkers for the identification of patients at risk for severe COVID-19, representing possible targets of intervention to limit COVID-19 severity [45,46,47].

The analyses of three large databases of individuals with immune-mediated inflammatory diseases have demonstrated an inverse association of anti-TNFα use and COVID-19-related outcomes [48], but further clinical trials are needed [49]. NF-kB pathway inhibition could be another potential therapeutic target for severe COVID-19 patients [50]. Its inhibition leads to the reduction of inflammatory cytokines and chemokines, such as *TNFα*, *IL-1*, *IL-6* and *MCP-1*, which are thought to be primarily involved in exuberant systemic inflammatory responses in COVID-19 patients [51]. It has been demonstrated that FBA, like its parental compound sodium butyrate, exerts an anti-inflammatory effect through NF-kB inhibition in DSS murine model [39]. Here, we demonstrated that FBA reduced Nf-kB activation in SARS-CoV-2-infected enterocytes.

NF-kB and Nfr2 are key pathways regulating the balance of cellular redox status and responses to stress and inflammation [52]. The genes under the control of Nfr2 protect against stress-induced cell death, and it has been suggested that Nfr2 are the master regulators of tissue damage during infection [53]. It has been demonstrated that Nfr2 overexpression inhibited NF-kB activation [54], and the expression of Nfr2-dependent antioxidant genes is significantly inhibited in COVID-19 patients [55].

Interestingly, a positive correlation between ACE2 and Nf-kB expression has been previously demonstrated, supporting the concept that targeting Nf-kB signaling can also inhibit ACE2 expression [56].

Major limitations of our study are related to the lack of experiments with small intestinal biopsies infected with the virus, due to the short survival time of these samples in culture, and with other target cells, i.e., the respiratory tract cells, and a lack of exploration into the other immune and non-immune factors involved in SARS-CoV-2 infection.

Our data need to be validated in more complex experimental settings, and clinical trials are advocated to further explore the potential of the FBA-based strategy against COVID-19.

In conclusion, we provided evidence on the protective action elicited by the butyrate releaser FBA against COVID-19. This molecule acts on at least two mechanisms: the reduction of the number of infected cells (through a down-regulation of the expression of the main mediators of the infection: ACE2, TMPRSS2 and NRP1), and the inhibition of the cytokine storm (through an inhibition of pro-inflammatory cytokine release mediated by Nfr2 activation and Nf-Kb inhibition).

Altogether, these results support the role of a healthy gut microbiome that can produce the right amount of butyrate to protect against SARS-CoV-2 infection, and support supplementing COVID-19 patients with beneficial microbial metabolites [43], with FBA as effective strategy against COVID-19.

## 4. Methods

### 4.1. Virus

Wild-type (WT) SARS-CoV-2 was isolated from the nasopharyngeal swabs of a patient with laboratory-confirmed COVID-19, as previously described [13]. Briefly, nasopharyngeal swabs in 2 mL of viral transport medium were collected for molecular diagnosis and frozen. Confirmed PCR-positive specimens were aliquoted and refrozen until virus isolation was initiated. Vero E6 cells were used for virus isolation from nasopharyngeal swabs. Vero E6 cells (8 × 10^5^) were trypsinized and suspended in DMEM, with 2% FBS in T25 flasks, to which 100 μL of the clinical specimen was added. The inoculated cultures were grown in a humidified 37 °C incubator with 5% CO_2_ and observed for cytopathic effects daily. When cytopathic effects were observed (7 days after infection), the cell monolayers were scrapped with the back of a pipette tip. The cell culture supernatant containing the viral particles was aliquoted (100 μL) and immediately frozen at −80 °C. Viral lysates were used for total nucleic acid extraction for confirmatory testing and sequencing. Experiments involving live viruses were performed in an accredited biosafety level-3 (BSL-3) laboratory following the methodologies represented in [13].

### 4.2. Human Enterocytes

We used a well-validated model of human enterocytes, the Caco-2 cells (American Type Culture Collection, Middlesex, UK; accession number: HTB-37). This cellular model has been recently used to explore SARS-CoV-2 infection and potential therapeutic strategies against COVID-19 [57,58]. The cells were cultured in high glucose Dulbecco’s modified Eagle medium (DMEM; Gibco, Berlin, Germany), supplemented with 10% fetal bovine serum (Sigma-Aldrich; St. Louis, MO, USA), 1% non-essential amino acids (Sigma-Aldrich; St. Louis, MO, USA), 1% (*v*/*v*) antibiotics (10.000 U/mL penicillin and 10 mg/mL streptomycin) (Euroclone Spa; MI, Italy) and 1% sodium pyruvate. Caco-2 cells were grown in an incubator at a temperature of 37 °C and CO_2_, 5%. The culture medium was changed every 2 days.

### 4.3. Ethics

The study protocol, the subject information sheet, the informed consent form, and the clinical chart were reviewed and approved by the Ethics Committee of the University of Naples Federico II (N.274/17/ESCOVID19). At the baseline, written and signed informed consent was obtained from all adult participants and from all parents/tutors of the minors. The study was conducted in accordance with the Helsinki Declaration (Fortaleza revision 2013), the Good Clinical Practice Standards (CPMP/ICH/135/95), the current Decree-Law 196/2003 regarding personal data and all the requirements set out in the European regulations on this subject.

### 4.4. Organ Culture of Mucosal Biopsies from Human Small Intestine

Small intestinal biopsies were collected via esophagogastroduodenoscopy (EGDS) from 5 healthy subjects (all Caucasian, 3 males, mean age 17 years, range 15–18 years), who had undergone an endoscopy for the presence of the following symptoms for at least 4 weeks: abdominal pain, constipation, diarrhea; and with the normal endoscopic appearance of the small intestine without histological inflammation. None of these subjects presented organic conditions.

Biopsies were used to perform organ culture experiments, as reported previously [59]. The biopsy culture was performed in RPMI-1640 medium (Sigma-Aldrich, Germany) without L-glutamine and supplemented with 10% fetal calf serum and antibiotic/antimycotic mixture (Gibco Invitrogen). Each sample was divided in two fragments for different conditions: medium alone (as the negative control) and treatment with FBA (2 mM). The fragments were placed on a stainless-steel mesh positioned over the central well of an organ culture dish, with the villous surface of the specimens uppermost. They were cultured for 24 h. Then, the specimens were immediately placed in RNAlater (Thermo Fisher Scientific, Waltham, MA, USA) and stored at −80 °C until analysis for RNA extraction.

### 4.5. Cell Treatment for Basal and Infection Conditions

**Figure 4:**
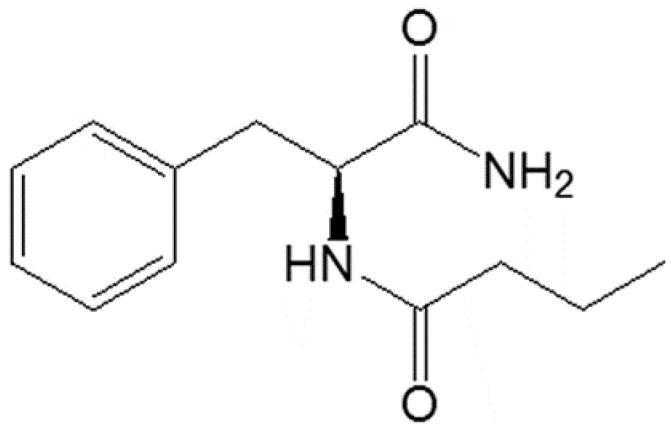
The molecular structure of *N*-(1-carbamoyl-2-phenyl-ethyl) butyramide (FBA).

For all experiments, a food grade batch of FBA, provided by Conagen Inc. (Bedford, Bedford, MA, USA), with 99.6% purity, was used. The molecular structure of FBA is depicted in Figure 4.

To investigate the effects of FBA in basal conditions, Caco-2 cells (2 × 10^5^ cells/well) were seeded in 6-well plates and were treated for 24 h with 2 mM FBA. Timing and dosing were selected based on the results of previous time-course and dose-response experiments performed at our laboratory. Then, the cells were washed with PBS, and harvested in TRizol for RNA extraction.

To explore the effects of FBA on the infection, Caco-2 cells were pretreated for 24 h with 2 mM FBA. Then, the cells were infected with 0.1 MOI of viral particles that belonged to the WT SARS-CoV-2 for 72 h. The cells were washed with PBS, and then harvested in TRizol or fixed in paraformaldehyde (PFA) for subsequent analysis. Non-infected Caco-2 cells were used as control (NI). All experiments were carried out in triplicate and for three times.

### 4.6. Infectivity Assays: Immunofluorescence Staining and Real-Time PCR for Nucleocapsid Protein

To determine the infectivity of SARS-CoV-2, Caco-2 cells were fixed with 4% PFA (Carlo Erba Reagents, MI, Italy) for 10 min at room temperature and processed for immunofluorescence staining to visualize viral antigen expression (Nucleocapsid protein, N). Autofluorescence due to free aldehyde groups from PFA treatment were blocked with 50 mM ammonium chloride (Sigma-Aldrich, St. Louis, MO, USA) in PBS for 10 min at room temperature. Cover slips were washed twice with PBS, and then cells were permeabilized with 0.1% Triton X-100 (PanReac AppliChem, Milan, Italy) in PBS for 10 min. After washing, the cells were blocked for 1 h using 1% BSA in PBS/0.2% Tween 20, and then incubated overnight at 4 °C with a specific primary antibody for Nucleoprotein (1:100; ProSci, 3851). Coverslips were washed with PBS and incubated with horseradish peroxidase (HRP)-conjugated goat anti-mouse (1:500; Alexa Fluor 594, A21203, Invitrogen, MA, USA) for 1 h at RT. Nuclei were stained with 4′6-Diamidino-2-phenylindole dihydrochloride (DAPI) (Invitrogen). Finally, cells were mounted with antifading Mowiol (Sigma-Aldrich). Cells were observed with a 63x objective on a Zeiss LSM980 confocal system equipped with an ESID detector and controlled by Zen blue software (Zeiss; Jena, Germany).

To detect the N gene in infected Caco-2 cells, total RNA was isolated with a TRizol reagent (Sigma-Aldrich), according to the manufacturer’s instructions. The RNA samples were quantified using the NanoDrop 2000c spectrophotometer (Thermo Scientific, Waltham, MA, USA) and RNA quality and integrity were assessed with the Experion RNA Standard Sense kit (Bio-Rad, Hercules, CA, USA). The RT-PCR for N of SARS-CoV-2 was performed with a Liferiver Novel Coronavirus (2019-nCoV) Real-Time kit (BioVendor, Brno, Czech Republic). These runs were performed using a 7900 Real-Time PCR System (Applied Biosystems, Carlsbad, CA, USA) under the following conditions: 45 °C for 10 min, 95 °C for 3 min, and 95 °C for 15 s and 58 °C for 30 s (×45 cycles).

The quantification cycle values of N are reported as means ± SD normalized to the internal control provided by the kit.

### 4.7. SARS-CoV-2 Molecular Mediators and Inflammatory Cytokine Analyses with Quantitative Real-Time PCR

A part of the RNA extracted (500 ng) was reverse transcribed in cDNA with a High-Capacity RNA-to-cDNATM Kit (Thermofisher, Waltham, MA, USA) according to the manufacturer’s instructions. Complementary DNA (cDNA) was stored at −80 °C until use.

To evaluate the effect on *ACE2*, *TMPRSS2* and *NRP1* gene expression, a quantitative real-time PCR (qRT-PCR) analysis was performed using a TaqMan Gene Expression Master Mix (Hs00174179_m1, Hs01122322_m1 and Hs00826128_m1, respectively; Thermofisher).

Inflammatory cytokines expression (*IL-6*, *IL-15*, *IL-1β*, *VEGFβ*, *TNF-α*, *MCP-1* and *CXCL1*) was evaluated using a SYBR green Master Mix (Applied Biosystems, Grand Island, NY, USA). The details of the primers used in these assays are provided in Table 1. The cDNAs were amplified using a 7900 Real-Time PCR System (Applied Biosystems) at the following cycling conditions: 50 °C for 2 min, 95 °C for 2 min, 40 cycles of 95 °C for 15 s and 60 °C for 1 min, followed by the melt curve setting of 1 cycle of 95 °C for 15 s, 60 °C for 1 min and 95 °C for 15 s.

Data analysis was performed using the comparative threshold cycle (CT) method and expressed as 2^-delta CT [25]. The beta-glucuronidase (GUSB) gene was used as the housekeeping gene (forward primer: 5′-GAAAATATGTGGTTGGAGAGCTCATT-3′; reverse primer: 5′-CCGAGTGAAGATCCCCTTTTTA-3′). Expression data were normalized to non-infected cells (NI). Each sample was analyzed in triplicate.

### 4.8. cDNA Library Construction for RNA Sequencing Analysis

To investigate the influence of FBA on the expression on the antiviral-related gene, another part of the RNA extract (1 µg) was used for RNA sequencing (RNA-Seq) analysis. The RNA sample quality was assessed with an Agilent 2100 Bioanalyzer RNA Nano chip device (Agilent, Santa Clara, CA, USA), and total RNA concentrations were determined using a NanoDrop ND1000 spectrophotometer (Nano-Drop, Wilmington, DE, USA). Agarose Gel Electrophoresis was also performed to test RNA degradation and potential contamination.

After the QC procedures, mRNAs were enriched using oligo(dT) beads. The mRNAs were mixed with the fragmentation buffer and broken into short fragments (~200 bp long). Then, the first strand of cDNA was synthesized with random hexamer primers. The second strand was synthesized using the SuperScript Double-Stranded cDNA Synthesis kit (Invitrogen, Camarillo, CA, USA) and was purified via magnetic beads. The ends were repaired and tailed with a single 3′ adenosine. Subsequently, the cDNA fragments were ligated to sequencing adapters. Quality controls of the obtained libraries were performed with Qubit 2.0 to test the library concentration preliminarily, Agilent 2100 to test the insert size and Q-PCR to precisely quantify the effective library concentration.

### 4.9. RNA-Seq Data Analysis

The analysis started with Illumina Paired End reads (FASTQ format) that were previously filtered by adapter sequences, with low-quality reads and with reads with more than 10% of unknown bases.

The quality assessment of cleaned FASTQ files was performed using FastQC (http://www.bioinformatics.babraham.ac.uk/projects/fastqc, accessed on 1 April 2014). Cleaned reads were of high quality; the percentage of bases with quality scores above 20 and 30 (Q20 and Q30) were 97.6 and 93.6 percent, respectively (Table 2).

Sequencing data were analyzed with the Tuxedo suite tool set (TopHat v2.0.14 and Cufflinks v2.1.0) following the analysis pipeline published in nature protocols 2012. TopHat is a fast splice junction mapper for RNA-Seq reads. It aligns RNA-Seq reads to the reference genome using the ultra-high throughput short read aligner Bowtie2 v2.2.6.0, and then analyzes the mapping results to identify the splice junctions between exons. TopHat was run with default options by providing the reference genome (assembly GRCh37/hg19, downloaded from UCSC and indexed with Bowtie2) and its related RefSeq reference transcriptome (downloaded from UCSC), along with the couple of FASTQ files (forward and reverse reads) for each sample. The alignment versus the reference genome (GRCh37/hg19 assembly) had an overall mapping rate of 92.49%, as shown in Table 2.

Next, Cufflinks was used to assemble the mapped reads (in the BAM file format) into possible transcripts and to generate a final transcriptome assembly. The tool Cuffmerge was exploited to merge transcriptomes from all samples and generate a common transcriptome file. Cuffdiff was finally used to detect differentially expressed genes and transcripts. It takes mapped reads from two or more biological conditions (provided as two or more biological replicates) and analyzes their differential expression of genes and transcripts, thus aiding in the investigation of their transcriptional and post transcriptional regulation under different conditions. The program was run by providing all the obtained BAM files (specifying the experimental condition and the replicate to which they belonged), the merged transcriptome assembly and the sequence of the reference genome. The software returned FPKM normalized counts (Fragments Per Kilobase of Exon Per Million Fragments Mapped) for each replicate and averaged FPKM counts for each experimental condition. Cuffdiff also assesses the statistical significance. Briefly, it performs a two-sided test for significance against a null hypothesis that the log-transformed ratio of the expression is unity (no change). The set of output files obtained by Cuffdiff was then inspected and explored using the R-Bioconductor package CummeRbund v2.16.0, which provides functions to read, subset, filter and plot results. Differentially expressed genes were those with a Log2 fold change above 0.5 or below −0.5 and an adjusted *p* value < 0.05. Among the differentially expressed genes, we focused on the antiviral pathways [40].

### 4.10. Western Blotting for Nf-kB and Nfr2 Protein Detection

Western blotting was performed on the total protein extracts of infected Caco-2 cells pretreated with FBA. For the total protein fraction, the harvested cells were washed in cold phosphate-buffered saline (PBS) and lysed in protein lysis buffer (RIPA). Protein concentrations in cell extracts were determined using the Bradford assay (BioRad, Milan, Italy). Thirty micrograms of total lysates were loaded onto 10% SDS-PAGE and then transferred to nitrocellulose membranes (ImmobilonR-Transfer Membrane, Tullagreen, Carrigtwohill, Co). The membranes were blocked with 5% non-fat milk in PBS, pH 7.6, 0.2% Tween 20 (PanReac AppliChem) and probed overnight at 4 °C with the specific primary antibodies for Nf-KB p65 (1:1000; Cell Signaling, MA, USA) and Nfr2 (1:1000, #89443, AbCam, Cambridge, UK). After washing in PBS, pH 7.6 and 0.2% Tween 20, the membranes were incubated with a horseradish peroxidase-conjugated goat anti-rabbit antibody (1:2000; Abcam). The immunoblots were visualized using ECL detection kits, with enhanced chemiluminescence (Pierce, Rockford, IL, USA). A mouse β-actin antibody (1:5000; Elabscience) was used as the control for equal loading of total lysates.

### 4.11. Statistical Analysis

The Kolmogorov–Smirnov test was used to determine whether variables were normally distributed. Descriptive statistics were reported as means and standard deviations (SDs) for continuous variables. To evaluate the differences among continuous variables, an independent sample *t*-test was performed. The level of significance for all statistical tests was two-sided, *p* < 0.05. All data were collected in a dedicated database and analyzed by a statistician using GraphPad Prism 7 (La Jolla, CA, USA).

## Figures and Tables

**Table 1:** Sequences of the primers to analyze pro-inflammatory cytokines.

Gene	Primer	Primer Sequence
*IL-6*	Forward	CTCGACGGCATCTCAGCC
	Reverse	GCCTCTTTGCTGCTTTCACAC
*IL-15*	Forward	CAGTTGCAAAGTAACAGCAATGAA
	Reverse	GCATCTCCGGACTCAAGTGAA
*IL-1β*	Forward	CTTTGAAGCTGATGGCCCTAA
	Reverse	CGCCATCCAGAGGGCAG
*VEGFβ*	Forward	AGAAGGAGGAGGGCAGAATCA
	Reverse	GATGGCAGTAGCTGCGCTG
*TNF-α*	Forward	CTTCTGCCTGCTGCACTTTG
	Reverse	TGATTAGAGAGAGGTCCCTGGG
*MCP1*	Forward	CTATAGAAGAATCACCAGCAGCAGCAAGT
	Reverse	TCTCCTTGGCCACAATGGTC
*CXCL1*	Forward	GCGCCCAAACCGAAGTCATA
	Reverse	ATGGGGGATGCAGGATTGAG

**Table 2:** Full description of RNA-Seq data analyses.

Sample ID	Analysis ID	Total Reads (M)	Read Length	Q20 (%)	Q30 (%)	Overall Mapping Rate (%)	Paired Mapping Rate (%)
NT1	NT_0	86.24	150	97.6	93.5	92.5	88.0
NT2	NT_1	85.33	150	97.7	93.7	92.6	88.1
NT3	NT_2	90.58	150	97.5	93.4	92.5	88.1
FBA1_2 mM	FBA_1_0	90.10	150	97.7	93.7	92.5	87.9
FBA2_2 mM	FBA_1_1	79.95	150	97.7	93.8	92.3	87.7
FBA3_2 mM	FBA_1_2	82.92	150	97.8	93.9	92.7	88.2

## Data Availability

The datasets used and/or analyzed during the current study are available from the corresponding author on reasonable request.

## Supplementary Materials

The following are available online, Table S1: Raw data of expression of genes related to anti-viral pathways regulated by FBA; Figure S1: Full-length gel of Phosfo-Nf-kBp65, Total Nf-kBp65 and β-actin; Figure S2: Full-length gel of Nrf-2 and β-actin.

## Sample Availability

Samples of the compounds are available from the authors.
