# Supplementary material for: A New Butyrate Releaser Exerts a Protective Action against SARS-CoV-2 Infection in Human Intestine
Source: Molecules. 2022 Jan 27;27(3):862. doi: 10.3390/molecules27030862 (PMC8838168; doi:10.3390/molecules27030862)

Supplementary Table

Title: A new butyrate releaser exerts a protective action against SARS-CoV-2 infection in human intestine

Lorella Paparo, Maria Antonia Maglio, Maddalena Cortese, Cristina Bruno, Mario Capasso, Erika Punzo, Veronica Ferrucci, Alessandro Lasorsa, Maurizio Viscardi, Giovanna Fusco, Pellegrino Cerino, Alessia Romano, Riccardo Troncone, Massimo Zollo.

Table S1. Raw data of expression of genes related to anti-viral pathways regulated by FBA

GeneSymbol	NT_0	NT_1	NT_2	FBA_0	FBA_1	FBA_2	Mean NT	Mean FBA	LOG2 FC	P-value	FDR
ADAM17	16,5619	20,3801	18,4302	37,2117	42,0147	42,101	18,4574	40,4424	1,13167	5,00E-05	9,04E-05
IRF7	13,8395	12,3931	12,2142	20,7526	16,2124	15,8258	12,8156	17,597	0,457425	5,00E-05	9,04E-05
HMGB1	95,3918	121,726	139,733	83,3335	94,8429	101,933	118,95	93,37	-0,349328	6,00E-04	0,00098927
CABIN1	7,5894	8,48003	6,47789	5,44999	6,42031	5,69417	7,51577	5,85482	-0,360296	4,00E-04	0,00067195
TLR2	2,4819	2,1279	2,33194	4,63643	4,64717	5,11941	2,31391	4,801	1,053	5,00E-05	9,04E-05
TMED7-TICAM2	48,964	45,8196	46,4385	150,625	120,319	125,396	47,074	132,113	1,48877	5,00E-05	9,04E-05
TRAF6	3,71262	3,89204	3,56438	13,8013	12,8793	13,8397	3,72301	13,5068	1,85914	5,00E-05	9,04E-05
CXCL11	0	0	0,018809	0	0,0292541	0	0,00626966	0,00975136	0,637215	1	1
CHUK	8,56507	8,56885	9,26262	12,7833	12,7622	14,8867	8,79885	13,4774	0,615157	5,00E-05	9,04E-05
TLR4	0,0429816	0,0593043	0,046403	0	0	0	0,049563	0	-0,0697887	1	1
IRAK1	37,4425	33,102	31,7843	19,8492	22,015	19,9548	34,1096	20,6063	-0,727089	5,00E-05	9,04E-05
CD14	5,00286	4,98943	5,03821	3,33784	3,1613	3,70884	5,01016	3,40266	-0,558195	5,00E-05	9,04E-05

Supplemental information

Title: A new butyrate releaser exerts a protective action against SARS-CoV-2 infection in human intestine

Lorella Paparo, Maria Antonia Maglio, Maddalena Cortese, Cristina Bruno, Mario Capasso, Erika Punzo, Veronica Ferrucci, Alessandro Lasorsa, Maurizio Viscardi, Giovanna Fusco, Pellegrino Cerino, Alessia Romano, Riccardo Troncone, Massimo Zollo.

Figure S1: Full-length gel of Phosfo-Nf-kBp65, Total Nf-kBp65 and β-actin.

Phosfo-Nf-kB p65 and β-actin **(A),** and Total Nf-kBp65 **(B)** were blotted on the same gel (PW: 65, 65 and 42 kDa, respectively). At left side of the image was represented the molecular weight markers (#G266, Opti-Protein XL Marker, Applied Biological Materials Inc. Richmond, Canada).

Starting at left side of the gel:

Lane 1: SARS-CoV-2 infected cells

Lane 2: FBA (butyrate releaser)

Lane 3: non-infected cells (NI)

(A)

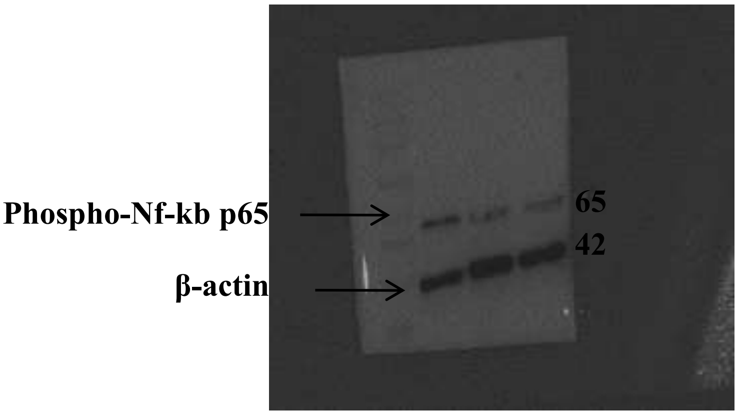

(B)

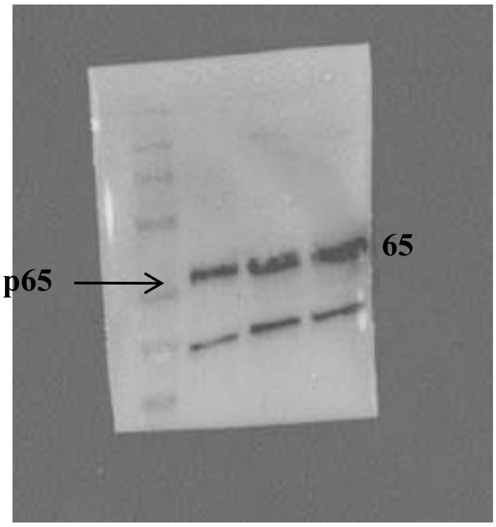

Figure S2: Full-length gel of Nrf-2 and β-actin.

Nfr2 **(A)** and β-actin **(B)** were blotted on the same gel (PW: 68 and 42 kDa, respectively). At left side of the image was represented the molecular weight markers (#G266, Opti-Protein XL Marker, Applied Biological Materials Inc. Richmond, Canada).

Starting at left side of the gel:

Lane 1: SARS-CoV-2 infected cells

Lane 2: FBA (butyrate releaser)

Lane 3: non-infected cells (NI)

Lane 4: represents a test sample for setting the procedure

(A)

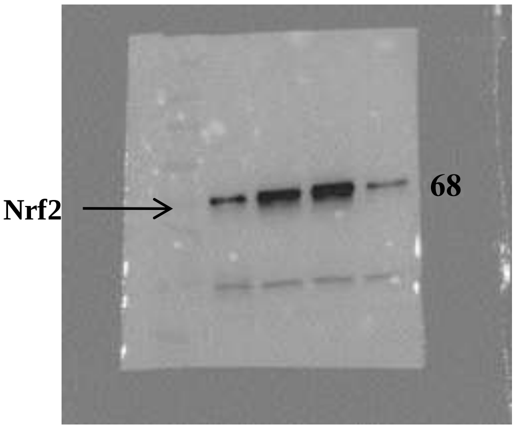

(B)

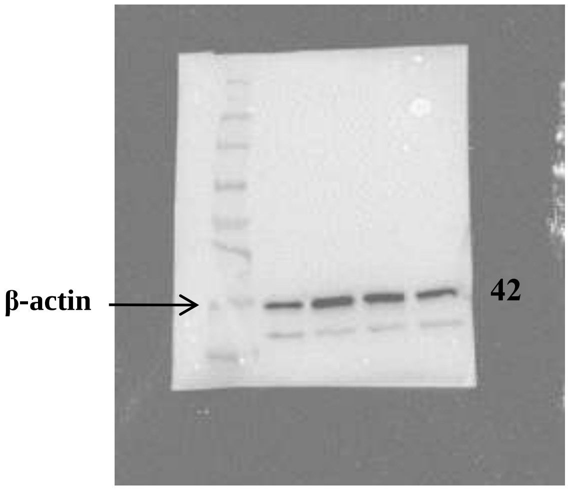

Supplement: Supplementary file 1 [file molecules-27-00862-s001.zip › molecules-1533588-supplementary.pdf]
